# A Novel Method to Compute the Contact Surface Area Between an Organ and Cancer Tissue

**DOI:** 10.3390/jimaging11030078

**Published:** 2025-03-06

**Authors:** Alessandra Bulanti, Alessandro Carfì, Paolo Traverso, Carlo Terrone, Fulvio Mastrogiovanni

**Affiliations:** 1 Department of Informatics, Bioengineering, Robotics, and Systems Engineering, University of Genoa, 16145 Genova, Italy; alessandro.carfi@unige.it (A.C.); fulvio.mastrogiovanni@unige.it (F.M.); 2 Department of Surgical and Diagnostic Integrated Sciences, University of Genoa, 16121 Genoa, Italy; paolotraverso@unige.it (P.T.); carlo.terrone@unige.it (C.T.); 3 Joint Research Lab on Interaction Technologies for Minimally Invasive and Open Surgery, IRCCS Policlinico San Martino, 16132 Genova, Italy; 4 IO Surgical Research Spin-Off, University of Genoa, 16126 Genova, Italy

**Keywords:** 3D segmentation, computed tomography, computer science, graphical user interface

## Abstract

The contact surface area (CSA) quantifies the interface between a tumor and an organ and is a key predictor of perioperative outcomes in kidney cancer. However, existing CSA computation methods rely on shape assumptions and manual annotation. We propose a novel approach using 3D reconstructions from computed tomography (CT) scans to provide an accurate CSA estimate. Our method includes a segmentation protocol and an algorithm that processes reconstructed meshes. We also provide an open-source implementation with a graphical user interface. Tested on synthetic data, the algorithm showed minimal error and was evaluated on data from 82 patients. We computed the CSA using both our approach and Hsieh’s method, which relies on subjective CT scan measurements, in a double-blind study with two radiologists of different experience levels. We assessed the correlation between our approach and the expert radiologist’s measurements, as well as the deviation of both our method and the less experienced radiologist from the expert’s values. While the mean and variance of the differences between the less experienced radiologist and the expert were lower, our method exhibited a slight deviation from the expert’s, demonstrating its reliability and consistency. These findings are further supported by the results obtained from synthetic data testing.

## 1. Introduction

The contact surface area (CSA), which refers to the region between the tumor and the surrounding unaffected organ, is of great importance in the surgical domain. It helps assess the complexity of the operation and aids in planning the surgical procedure. This indicator was proposed by Leslie et al. [1] in 2014 in the field of urology and has been increasingly studied since then, as it is closely correlated with various peri-operative outcomes specifically for partial nephrectomy, a surgical procedure in which only the tumor is removed from the kidney, preserving as much healthy tissue as possible. The CSA is a key metric in this context, as it helps assess the complexity of tumor resection and aids in surgical planning. Moreover, the CSA is not only essential for defining resection boundaries but it also serves as a strong predictor of intraoperative and post-operative parameters [2,3], offering valuable guidance for surgical planning and optimizing patient outcomes. Therefore, identifying precise methods for calculating the CSA is of utmost importance.

Few methods have been proposed for computing the CSA between the kidney and the tumor; however, they present certain limitations, as they rely on manual annotations and calculations performed by operators, along with approximations concerning tumor geometry. As previously mentioned, the first method for CSA estimation was proposed by Leslie et al. (2014) [1]. This approach involved approximating the tumor as a sphere and multiplying its total surface area by the percentage of intraparenchymal components (i.e., portions of the tumor extending into the kidney parenchyma), which was measured automatically using a 3D shape reconstruction technique. Two years later, Hsieh et al. (2016) [4] proposed an approach to estimate the CSA. This proposal aimed to correct Leslie’s approach, which mistakenly assumed a direct relationship between the CSA and intraparenchymal percentage. The technique introduced by Hsieh et al. (2016) [4] for CSA computation preserves the spherical assumption for tumors and adopts the formula 2×π×r×d, where *r* is the maximum radius of the tumor, and *d* is the maximum depth of the tumor’s intrusion into the uninvolved parenchyma. According to this method, these two quantities are extracted from visual inspection of the sagittal or coronal planes of Digital Imaging and Communications in Medicine (DICOM) images obtained from computed tomography (CT) or Magnetic Resonance Imaging (MRI). Subsequent studies have utilized the formula and the method introduced by Hsieh et al. (2016) [4], analyzing 2D images to identify the maximum radius and depth, as well as aiming to assess the predictive capacity of the CSA for peri-operative parameters [2,3,5,6,7].

More recently, due to advancements in medical imaging techniques, which made widely available software tools for the 3D reconstruction of organs, surgeons have shifted away from visual image analysis methods and begun to measure the CSA through 3D reconstructions of both kidneys and tumors. Takagi et al. (2019) [8] and Bianchi et al. (2022) [9] evaluated the contact area by manually outlining the boundary of the tumor on the kidney reconstructed in 3D. Meanwhile, Umemoto et al. (2023) [10] utilized a technique based on 3D reconstructions, enabling tumor removal simulation and the estimation of the corresponding CSA. However, these studies rely on human intervention to annotate the CSA on the 3D reconstruction of the organ or tumor, resulting in subjective estimates. Finally, a study by Wood et al. (2024) [11] proposed a deep learning technique for organ–tumor segmentation and CSA extraction from the reconstructed models. However, the specific details of their method are not provided, and no source code has been released.

To address the limitations of previous CSA computation methods, we propose a novel approach that, starting from 3D reconstructions of the tumor and the organ, computes the CSA in a fully automated manner. Unlike previous techniques that rely on geometric assumptions or manual measurements, our method accurately captures the contact surface area by analyzing the detailed surface geometry of both the organ and the tumor. This eliminates observer-dependent variability and ensures a more objective and reproducible estimation of the CSA. Specifically, our work aims to introduce a new technique for CSA estimation that improves precision and objectivity. Since we recognize that CSA computation heavily depends on the quality of 3D reconstruction and that deriving 3D models from CT scans is not always straightforward, we propose an experimental protocol to ensure the correct segmentation of organs and tumors. Additionally, the proposed algorithm has been designed to be robust to 3D model imperfections caused by the reconstruction process.

To evaluate the accuracy of our approach, we tested it on both a synthetic benchmark with known ground truth values and real clinical data from patients undergoing partial nephrectomy. Our findings demonstrate that our method provides consistent and reliable CSA measurements, significantly reducing the need for human annotators.

This article presents the segmentation protocol adopted for reconstructing the 3D models of kidneys and tumors from CT scans, provides an in-depth description of the algorithm, and reports a quantitative evaluation on a synthetic dataset, as well as both qualitative and quantitative evaluation on patient reconstruction data—82 patients for the former and 78 for the latter. The algorithm implementation and a simple graphical user interface are publicly available to increase the likelihood of reproducing our work and promote the adoption of our technique.

## 2. Materials and Methods

### 2.1. Segmentation Protocol and Procedure

Our approach to computing the CSA relies on 3D models of the organ and the tumor. Therefore, the quality of the 3D reconstruction is crucial to ensure consistency in the CSA computation. The quality of the reconstructions depends primarily on the accuracy of segmentation. Correctly scanning the region of interest and performing precise operations for the segmentation contributes to obtaining a more accurate reconstruction. Additionally, the resolution of CT images plays a fundamental role as well. High-resolution images contain fewer artefacts, enabling more precise recognition of various regions of interest.

Currently, a standard fully automated segmentation algorithm for the kidney does not exist, although a few preliminary approaches have been proposed so far [11,12,13,14]. Indeed, this task is challenging due to the irregular structure of the kidney, which can vary significantly from person to person. Moreover, the presence of low contrast in some cases can introduce artifacts, hindering the precise reconstruction of the kidney structure. Finally, it is difficult to precisely identify kidney contours because they are scattered into various layers in the tomographic images [12,15].

**Figure 1:**
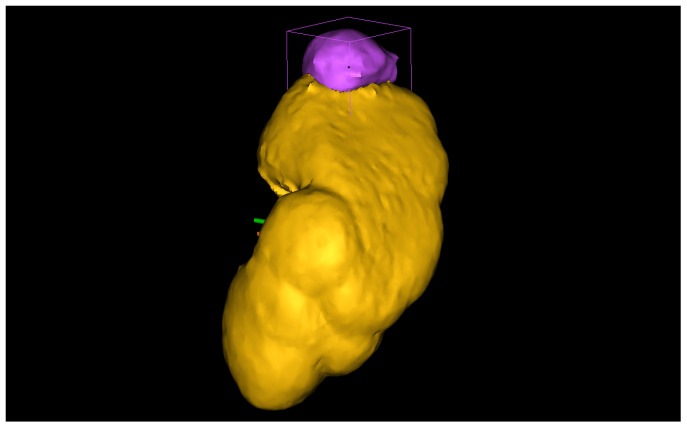
A 3D reconstruction of a kidney, in yellow, and a tumor, in purple.

In this Section, we outline the segmentation protocol for obtaining accurate 3D reconstructions of organs and tumors. Our protocol is specific for the reconstruction of kidneys and their associated tumors. The main objective of the protocol is to describe how a human operator should proceed to segment the kidney and the tumor to ensure an accurate reconstruction. Therefore, we structured the protocol into three steps: kidney segmentation, tumor segmentation, and reconstruction refinement. After completing these steps, accurate 3D reconstructions were generated (see Figure 1) and exported as STL files. These files were then provided as input to our algorithm for calculating the CSA.

Our 3D reconstructions use CT DICOM images, which enable the visualization of the human body in three different planes, i.e., axial, coronal, and sagittal. In our implementation, we adopted Materialise Mimics InPrint (https://www.materialise.com/en/healthcare/mimics-inprint, accessed on 3 March 2025) from Materialise NV. This choice does not impose any contingent limitation on our segmentation protocol, which can be applied to other scenarios and based on other medical-grade software applications.

*Kidney segmentation*. This step requires both automated and manual operations by the human operator. First, the operator must navigate the CT scan to identify the kidney. Then, the operator has to define the region corresponding to the kidney by manually selecting the appropriate radio density range. Points with radio density within the selected range will be considered part of the kidney. In Materialise Mimics InPrint, this procedure can be performed using the *Threshold* functionality and setting an appropriate *Hounsfield Unit* (HU) based threshold. HU is the scale used to describe quantitatively the radio density information within an image. This value, computed from the absorption coefficient of the material under standard conditions, enables us to distinguish between different tissues and structures based on their chemical composition.

**Figure 2:**
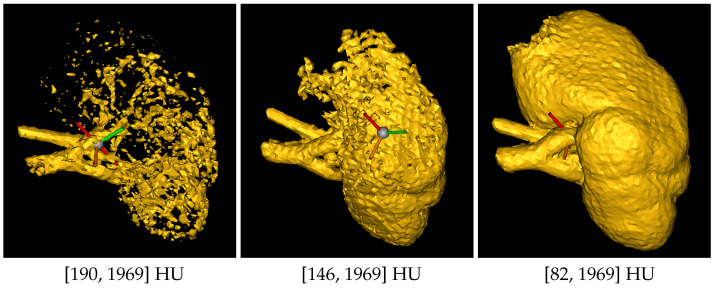
It can be observed that as the HU lower bound varies, the reconstruction accuracy differs. In particular, from **left** to **right**, there is a decrease in the lower bound, resulting in a more defined and precise reconstruction of the kidney.

**Figure 3:**
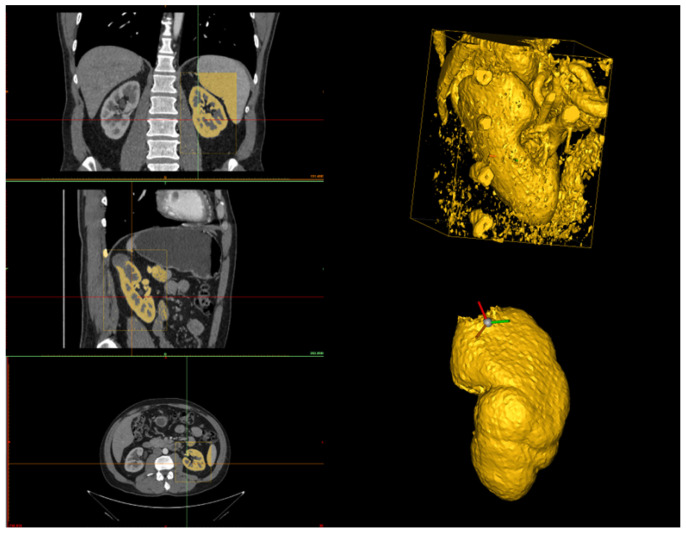
On the **left-hand** side, the selection of the region of interest in the three planes (axial, coronal, and sagittal) is shown, where the region defined in yellow represents the kidney based on a threshold application. On the **top right-hand** side, the 3D reconstruction after the threshold application is displayed. On the **bottom right-hand** side, the 3D reconstruction after manual cleanup, where all surrounding structures have been removed, leaving only the kidney.

The human operator can set the HU range by choosing upper and lower bounds to highlight all the kidney structures and minimize cavities and holes in the kidney model (see Figure 2). Since the quality of the CT scan significantly influences the HU range, it is difficult to define a universal range. However, based on our experience with high-quality CT scans, the recommended range is (80–1969) HU. In the case of lower-quality scans, these limits can be adjusted to achieve an optimal reconstruction. Additionally, after setting these values, it is advisable to retain only large regions and automatically fill small holes. Once the segmentation of the region of interest (that is, a bounding box) is performed, the result is the 3D model of the kidney, together with other smaller parts from the surrounding tissues (see Figure 3).

Any unrelated kidney components can be manually removed easily. This result can be achieved by using a specific command for deletion, allowing these parts to be removed directly from the 3D reconstruction or the 2D images. In the first case, this is performed by selecting the part to be removed, while in the second case, this is done by deleting it slice by slice until it is eliminated.

**Figure 4:**
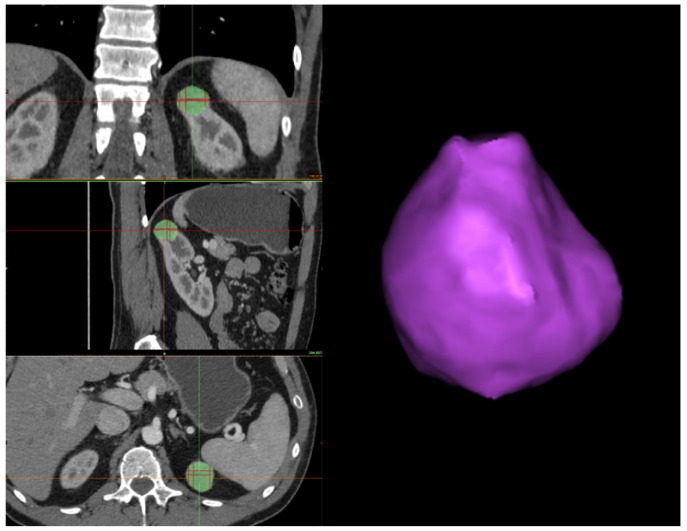
On the **left-hand** side, the automated 3D interpolation of the tumor is shown based on the manually drawn silhouette in the three planes, where the green region represents the tumor in each plane. On the **right-hand** side is the 3D reconstruction of the tumor obtained from the interpolation of the three axes.

*Tumor segmentation*. In this stage, similar to kidney segmentation, a hybrid approach is employed, which encompasses semi-automatic processes executed through software tools and manual interventions performed by a human operator. Nevertheless, the segmentation process for tumors differs from that of kidneys. In this case, the tumor contour is directly outlined. Initially, the human operator must identify the tumor in all three sections (i.e., axial, coronal, and sagittal) from the CT scan. Subsequently, the tumor’s boundary must be outlined on each section using the *3D interpolate* tool available in Materialise Mimics Inprint. Specifically, when delineating the tumor’s boundary, Materialize uses an algorithm that semi-automatically segments the tumor over the various slices and produces a 3D reconstruction of it, which may change depending on the selection of the tumor boundary in the next slice (see Figure 4).

**Figure 5:**
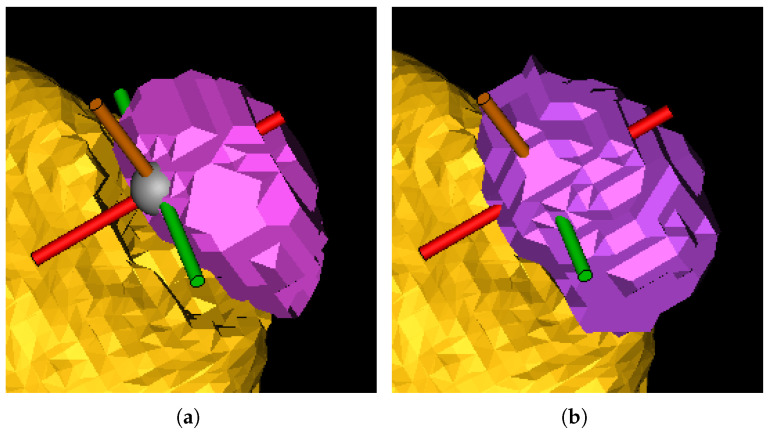
An example of the importance of correctly outlining the tumor perimeter. In the left image (**a**), the reconstructed tumour exhibits a missing part, leading to a gap between the tumor and the kidney. The right image (**b**) depicts the tumor after the missing part has been manually added.

After the automated reconstruction, the human operator should manually inspect the model. Missing or extra sections should be corrected manually (see Figure 5).

*Reconstruction refinement*. Since the organ and the tumor are reconstructed independently, it is essential to check for pairwise inconsistencies. These could involve irregularities in the kidney’s boundary, holes in the kidney interface with the tumor, overlapping, or a lack of contact between the two.

**Figure 6:**
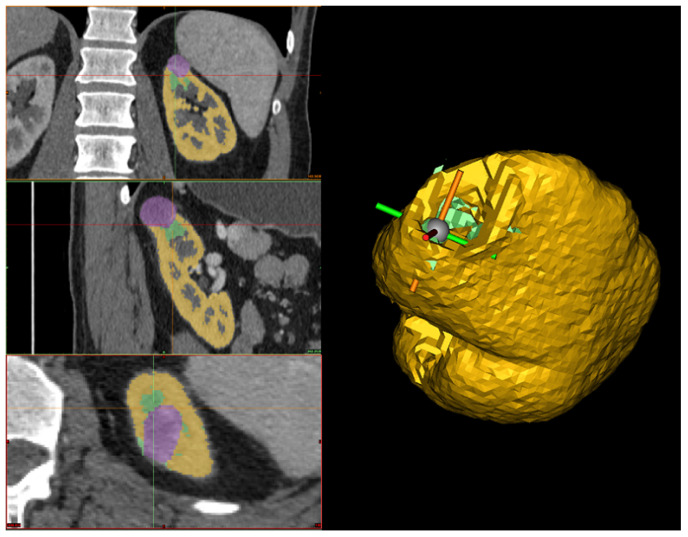
On the **left**, the hole-filling process across the three different planes is shown, where the purple represents the tumor, the yellow represents the kidney, and the green indicates the manually filled region used to close the gap between the tumor and the kidney. On the **right**, the 3D reconstruction of the kidney after the filling process is displayed, where the yellow represents the kidney, and the green corresponds to the filled part of the kidney.

Therefore, the first step in correcting the reconstruction is to remove any tumor parts mistakenly considered part of the organ during kidney segmentation. Then, the human operator should inspect the interface between the tumor and the kidney to confirm the absence of gaps. If any holes are present, the *Fill* command is used to address them while checking each slice across the three sections (see Figure 6). Finally, before exporting the 3D models, a smoothing operator is applied to slightly refine the geometries of the two volumes. The final result can be seen in Figure 1, which was obtained from a combination of automated and manual tumor and organ segmentation. As previously discussed, organ and tumor segmentation is fundamental for the algorithm presented in Section 2.2. Here, we described a manual procedure for this segmentation. However, in the future, this could be replaced with a fully automated process.

### 2.2. Algorithm

The approach presented in this Section can estimate the CSA between an organ and a tumor using their 3D models, which were reconstructed as described in the previous Section. All the formulas and the algorithm listed below can also be found in the Supplementary file. For our approach to be effective, these models should be accurate, non-hollow, and maintain their relative poses. Although our approach is introduced here for computing the CSA between an organ and a tumor, the algorithm can be applied to any couple of 3D objects as long as they satisfy the previously mentioned requirements. In 3D computer graphics, the shape of an object is defined by a polygonal mesh PM, typically composed of triangular faces *F*. The number *N* of faces depends on the mesh complexity:

(1) $$PM=\{F_{1}\cdots F_{j}\cdots F_{N}\}.$$

A face, according to its shape, is characterized by a set of *M* vertices, such as

(2) $$F_{j}=\left\{V_{j1}\cdots V_{jk}\cdots V_{jM}\right\},$$

where each vertex is defined in the 3D Cartesian space as

(3) $$V_{jk}=\left\{V_{jk}.x,V_{jk}.y,V_{jk}.z\right\}.$$

Our approach for computing the CSA between two objects, $O_{1}$ (for example, the organ) and $O_{2}$ (for example, the tumor), is based on the following conceptual position. We assume that each object $O_{i}$ with i∈[1,2], is described by a $PM_{i}$, which is composed of $N_{i}$ planar faces as

(4) $$PM_{i}=\left\{F_{i1},\cdots F_{ij},\cdots F_{iN_{i}}\right\}.$$

Each face $F_{ij}$ is described by a ordered set of vertices of dimension $M_{ij}$

(5) $$F_{ij}=\left\{V_{ij1}\cdots V_{ijk}\cdots V_{ijM_{ij}}\right\},$$

and each vertex is expressed in 3D Cartesian coordinates.

Our algorithm requires six intermediate steps. The first three involve the definition and the computation of intermediate elements:

1. *Computation of the centroids*: The centroids of each face in the mesh composing an object are computed. This procedure is run for both the kidney and the tumor.
2. *Computation of the centroid-to-centroid distance*: For each centroid of the smaller object (typically, but not necessarily, the tumor), we calculate the distance to the closest centroid belonging to the other object.
3. *Computation of the threshold*: We compute a threshold, which we use to classify centroids as belonging to the CSA, by analyzing the distribution of centroid-to-centroid distances.

The last three steps involve the definition, correction, and computation of the CSA:

4. *Definition of the CSA*: We select the distances computed in Step 2 that are smaller than the threshold to obtain the list of faces belonging to the CSA.
5. *Refinement of the CSA*: We inspect if there are disconnected parts of the mesh in the CSA, and, in such cases, we determine if they belong to the CSA or not.
6. *Computation of the CSA*: We calculate the CSA by summing up the areas of all the faces composing it.

#### 2.2.1. Computation of the Centroids

Our method acts upon the mesh faces. However, since we do not make any assumptions about the number of vertices composing each face (in principle, not all faces may have the same number of vertices), we need to extract a coherent descriptor for each face whose structure is independent of the number of vertices. Therefore, we selected the face centroid $C_{ij}$ as a descriptor, and we computed it for each face of both meshes using the following procedure:

  1: **for** each $PM_{i}$ ∈ {$PM_{1}$, $PM_{2}$} **do**
  2:       **for** *j* = 1 to $N_{i}$ **do**
  3:             x←0
  4:             y←0
  5:             z←0
  6:             **for** *k* = 1 to $M_{ij}$ **do**
  7:                      $x\leftarrow x+V_{ijk}.x$
  8:                      $y\leftarrow y+V_{ijk}.y$
  9:                      $z\leftarrow z+V_{ijk}.z$
10:             **end for**
11:             $C_{ij}.x\leftarrow x/M_{ij}$
12:             $C_{ij}.y\leftarrow y/M_{ij}$
13:             $C_{ij}.z\leftarrow z/M_{ij}$
14:       **end for**
15: **end for**

In this step, the centroid 3D coordinates (that is, *x*, *y*, and *z* coordinates) are calculated for each mesh face. Each coordinate is determined as the sum of the corresponding coordinates of each vertex (lines 6–10) composing the considered face, which is divided by the total number of vertices (lines 11–13).

#### 2.2.2. Computation of the Centroid-to-Centroid Distance

To determine which faces belong to the CSA, as a feature, we use the distance between pairwise faces belonging to the two objects. At this stage, we compute the pairwise distances between each face in the mesh of the smaller object and the closest face belonging to the other object. The distance *d* between two faces is computed as the Euclidean distance between the centroids of the two faces, irrespective of their pose. The result of this procedure is saved in a vector structure $D=d_{1},\cdots ,d_{L}$, where $L=min(N_{1},N_{2})$.

  1:
$p\leftarrow argmin(N_{1},N_{2})$

  2:
$q\leftarrow argmax(N_{1},N_{2})$

  3: **for** *i* = 1 to $N_{p}$ **do**
  4:       **for** *j* = 1 to $N_{q}$ **do**
  5:             $Dt_{j}\leftarrow euclidean\_distance\left(C_{pi},C_{qj}\right)$
  6:       **end for**
  7:       $D_{i}\leftarrow min\left(Dt\right)$
  8: **end for**

In lines 1–2, we determine which of the two PMs has the maximum number of faces and which has the minimum. In lines 3–6, we calculate the distances between each face of the smaller PM and all the faces of the other PM. Then, at line 7, we select only the minimum distance. This results in a vector containing, for each face, only the minimum distances.

#### 2.2.3. Computation of the Threshold

**Figure 7:**
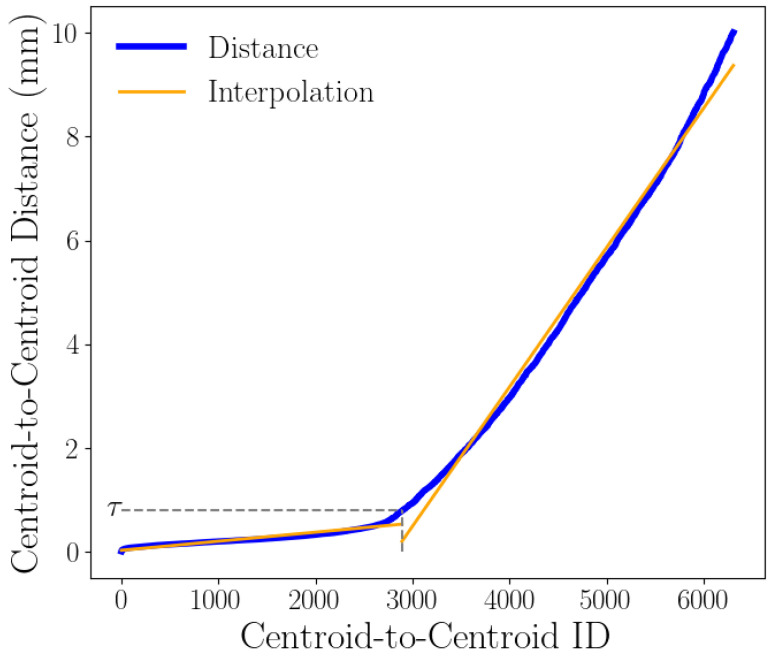
A visual representation of a threshold-finding process. The blue line shows the distribution of centroid-to-centroid distances, while the orange lines represent the closest approximation to the distribution of distances.

As stated previously, our approach aims to identify which faces belonging to the smaller mesh are part of the CSA. As we will see in Section 2.2.4, this can be achieved using a classification feature defined as a threshold τ applied on the centroid-to-centroid distance, as computed in Section 2.2.2. However, due to variations in the distribution of distances between different object pairs and the likelihood of imperfections in the 3D reconstruction, a static threshold is not appropriate. We have devised a method to obtain the threshold for each object pair by ordering the distance vector *D* and analyzing the distribution of the distances therein. Figure 7 provides a visual representation of this process.

As shown in the figure, the ordered distances are low on the left-hand side and increase with an evident discontinuity. Such a discontinuity results from the transition between faces that belong to the CSA and those that do not. We determined the threshold value by identifying the two lines that best approximate the distance distribution and finding their intersection point. This result can be achieved by dividing the ordered distances into two parts and performing linear interpolations on both. For the linear interpolation, we adopted a least square polynomial approach. We varied the division point over all the samples in the distance vector to find the best division point and the resulting threshold. To improve precision and narrow the search space, we focused the threshold search on a subset of the sorted distances, specifically those under 1 cm. This value was chosen based on the assumption that the CSA point should be approximately at zero distance. Therefore, anything over 1 cm is definitely not part of the CSA.

  1:
Dt←quicksort(D)

  2:
F←0

  3: **for** *i* = 1 to *L* **do**
  4:       **if** $Dt_{i}<1$ cm **then**
  5:             $Ds_{i}\leftarrow Dt_{i}$
  6:             F←F+1
  7:       **else**
  8:             break
  9:       **end if**
10: **end for**
11: **for** *i* = 2 to F−1 **do**
12:       $f1\leftarrow lsq\_fit(Ds_{\left[1,i\right]})$
13:       $f2\leftarrow lsq\_fit(Ds_{\left[i+1,F\right]})$
14:       ${\bar{D}}_{\left[1,i\right]}\leftarrow f1\left(1,i\right)$
15:       ${\bar{D}}_{\left[i+1,F\right]}\leftarrow f2\left(i+1,F\right)$
16:       $d_{i}\leftarrow 0$
17:       **for** *j* = 1 to *F* **do**
18:             $d_{i}\leftarrow d_{i}+euclidean\_distance\left(Ds_{j},{\bar{D}}_{j}\right)$
19:       **end for**
20: **end for**
21:
id=argmin(d)

22:
$\tau =Ds_{id}$

In lines 3–5, we select the distance values less than 1 cm. In lines 11–15, we perform the interpolation. Then, in lines 16–19, we compute the point-to-point distances between the actual distance values and the interpolated ones, measuring the cumulative error for each case. In line 21, we choose the value with the minimum cumulative error as the one identifying the threshold.

#### 2.2.4. Definition of the CSA

Once the threshold τ is identified, it is possible to determine which faces of the smaller object are part of the CSA. This result can be obtained by selecting those faces with a centroid-to-centroid distance lower than the identified threshold. The outcome of this phase is a list of the faces belonging to the CSA.

  1:
IDs←{}

  2:
j←1

  3: **for** *i* = 1 to L **do**
  4:       **if** $D_{i}<\tau$ **then**
  5:             $IDs_{j}\leftarrow i$
  6:             j←j+1
  7:       **end if**
  8: **end for**

In line 4, we check if the distances computed in Section 2.2.2 are less than the threshold value. When this condition is met, the ID is inserted in the list (line 5). The output is a list containing all the IDs of faces belonging to the CSA.

#### 2.2.5. Refinement of the CSA

**Figure 8:**
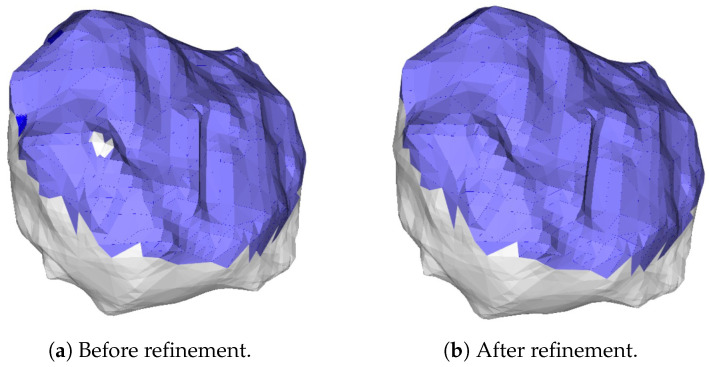
On the **left-hand** side (**a**), the *contact surface area* (CSA) is displayed over a tumor before refinement. It can be noticed that the used threshold divides the original mesh into three partitions. On the **right-hand** side (**b**), the CSA after refinement shows that one of the two surfaces, which was considered non-contact at the beginning, has been associated back to the CSA.

The CSA definition classifies the faces of the smaller mesh into two categories: in-contact and non-in-contact. Nevertheless, due to imprecision in the 3D reconstruction process, instances may arise where the CSA policy erroneously divides the original mesh into more than two segments, as illustrated in Figure 8 on the left side. In such scenarios, the in-contact mesh remains singular while multiple non-in-contact meshes emerge. Consequently, our approach includes a verification step to identify cases with more than two meshes and refines the CSA to address this discrepancy if required.

To refine the CSA, the mesh obtained by removing the CSA faces is inspected. If the resulting mesh is partially connected, nothing is done. However, if the resulting mesh contains a set of disconnected meshes, these meshes are processed to determine whether they should belong to the CSA. First, all the vertices of the faces that are not part of the CSA are added into a graph *G* (line 4). It is checked whether the graph is partially connected. If the graph is partially connected, nothing is done. Otherwise, connected sub-graphs are identified, and the corresponding face IDs are extracted (line 6).

  1: **for** *i* = 1 to *L* **do**
  2:       **if** i∉IDs **then**
  3:             **for** *k* = 1 to $M_{pi}$-1 **do**
  4:                   *G.add_edge*($V_{pik}$, $V_{pi(k+1)}$)
  5:             **end for**
  6:             *G.add_edge*($V_{piM_{pi}}$, $V_{pi0}$)
  7:       **end if**
  8: **end for**
  9: **if** *not isconnected(G)* **then**
10:       Sm←connected_components(G)
11: **end if**

This procedure identifies a list of sub-meshes named Sm (line 10). The list length is equivalent to the sub-meshes identified by the *connected_components* method. Each element in the list is itself a list containing all the IDs of the faces that constitute that particular sub-mesh. At this point, we need to determine which sub-meshes should be considered part of the CSA. If, after removing the CSA, we have more than one sub-mesh (that is, |Sm|>1), it means that the remaining sub-meshes contain points that the threshold mechanism wrongly labeled as not belonging to the CSA. Therefore, for each sub-mesh, we looked for the faces with the highest centroid-to-centroid distance $D_{i}$, and we picked the mesh with the furthest face as the one representing the non-contact surface. All the other sub-meshes’ faces were added to the CSA, as shown in Figure 8 on the right-hand side.

  1:
t←{0}

  2: **for** *i* = 1 to |Sm| **do**
  3:       **for** *j* = 1 to $|Sm_{i}|$ **do**
  4:             **if** $t_{i}\leq D_{Sm_{ij}}$ **then**
  5:                   $t_{i}\leftarrow D_{Sm_{ij}}$
  6:             **end if**
  7:       **end for**
  8: **end for**
  9:
not_csa=argmax(t)

10: **for** *i* = 1 to |Sm| **do**
11:       **if** j≠not_csa **then**
12:             **for** *j* = 1 to $|Sm_{i}|$ **do**
13:                   k←length(IDs)
14:                   $IDs_{k+1}\leftarrow j$
15:             **end for**
16:       **end if**
17: **end for**

In lines 2–8, we select for each sub-mesh the face with the highest distance to the CSA, and in line 9, we designate the one with the maximum distance as the surface not in contact. In lines 10–17, we add the faces IDs from the other sub-meshes to the list of faces IDs belonging to the CSA.

#### 2.2.6. Computation of the CSA

After ensuring that the IDs of all the faces belonging to the CSA are stored in the IDs vector, the overall area of the CSA can be computed. To do this, we sum up all the face areas, as shown below (lines 2–5):

  1: CSA←0
  2: **for** *i* = 1 to |IDs| **do**
  3:       $j\leftarrow IDs_{i}$
  4:       $CSA\leftarrow CSA+area(C_{pj})$
  5: **end for**

Since we aimed at preserving generality, we made no assumptions about the shape of mesh faces. While most meshes consist of triangular faces, we chose to remain agnostic to this characteristic. To calculate the area of each face, we converted the vertices of each of them from 3D to 2D by projecting them onto the plane of the faces. Then, to compute the total area, we used the Shoelace formula as

(6) $$A=\frac{1}{2}\left|\sum_{i=1}^{n-1}x_{i}y_{i+1}+x_{n}y_{1}-\sum_{i=1}^{n-1}x_{i+1}y_{i}-x_{1}y_{n}\right|,$$

where *A* represents the area of the polygon, *n* represents the number of vertices of the polygon, and *x* and *y* are the coordinates of the vertices in the face plane.

## 3. Results

**Figure 9:**
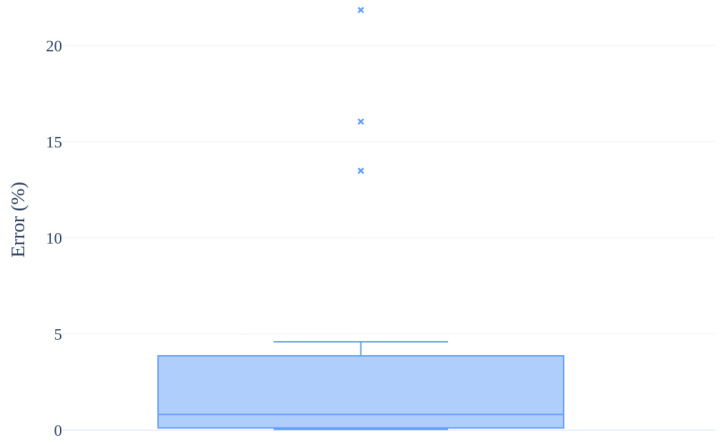
Percentage errors observed in the synthetic benchmark, with three outliers marked with a cross.

To evaluate the accuracy of our approach, we used a set of synthetic models. We created these models using the CAD software Fusion 360, allowing us to have a ground truth of the contact surface area. The synthetic benchmark contains 20 pairs of models and is publicly available on the GitHub 2.48.1 repository (https://github.com/ACarfi/contact-surface-area-gui, accessed on 3 March 2025). The synthetic organ is always a rectangular base prism with a cut corresponding to the synthetic tumor, while the synthetic tumor varies from a simple sphere to more complex shapes. The box plot in Figure 9 depicts the evaluation results, expressed as a percentage error. As can be seen from the box plot, except for a few outliers, the system demonstrated low percentage errors, with a median percentage error close to zero.

**Figure 10:**
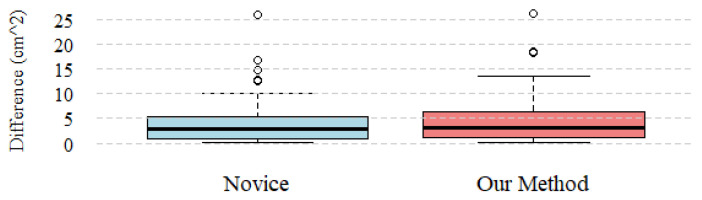
The blue boxplot on the **left** illustrates the distribution of differences between the measurements taken by the experienced and less experienced radiologists. On the **right**, the coral-colored boxplot represents the distribution of differences between the algorithm’s measurements and those of the experienced radiologist.

We also conducted both qualitative and quantitative tests on real tumor and organ reconstructions. We reconstructed 87 organ–tumor pairs from 82 patients who underwent partial kidney nephrectomy and processed the resulting 3D models using our algorithm. The reconstructions of these organs and tumors were carried out following the procedure proposed in Section 2.1. To ensure a sufficiently accurate result, the process for each kidney–tumor pair took an average of one hour of work. After visually inspecting the computed CSA, the outcomes for each of the 87 pairs were deemed qualitatively acceptable. Additionally, we performed quantitative measurements of the CSA values in 78 patients—those with only one tumor. On the same patients, the CSA was also calculated using Hsieh’s method. This method was applied in a double-blind manner by two radiographers with different levels of experience: one with 20 years of experience, i.e., expert, and the other with 3 years, i.e., novice. Given the absence of ground truth values, we adopted the CSA values calculated by the most experienced radiologist as the closest approximation to the truth. First, we assessed the normality of the value distributions using the Shapiro–Wilk test. The distributions of the three CSAs evaluated by the three methods—two from Hsieh et al. (2016) [4] and one from the algorithm—were all found to be non-normal (*p*-value < 0.05). Next, we used Spearman’s correlation coefficient to evaluate the agreement between the measurements of the novice and the expert (ρ = 0.93), as well as between the algorithm’s measurements and those of the expert (ρ = 0.92). We also compared the CSA distributions between the expert and the novice, as well as between the expert and the algorithm, using a boxplot to visualize the distribution (Figure 10). The median difference between the expert’s and the novice’s measurements was 2.97 cm^2^, while the median difference between the expert’s measurements and our approach was 3.08 cm^2^. The mean and variance of the differences between the two Hsiesh’s measurments were 3.92 cm^2^ and 18.36 cm^4^, respectively, while the mean and variance between the expert’s measurements and those of our algorithm were 4.43 cm^2^ and 21.55 cm^4^. Additionally, the algorithm also computed the total tumor area, and for all 87 tumors, these values matched with those automatically calculated by the Materialise Mimics software 3.0.

**Figure 11:**
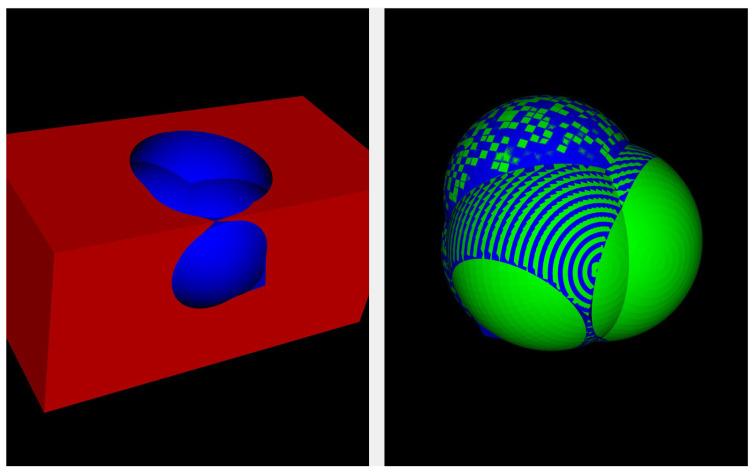
Our graphical user interface displays an example of the synthetic benchmark and the resulting CSA analysis. The image on the left shows an example of the synthetic kidney and synthetic tumor benchmarks created for the CSA calculation, while the image on the right shows the blue part of the synthetic tumor representing the CSA computed by the algorithm.

The solution described in the previous Section has been developed into a Python 3.12.9 class and integrated into a graphical user interface (GUI) for ease of use. Due to the limitations of the libraries used to manage the STL files, the current implementation only supports triangular meshes. However, the code has been written to preserve the solution’s nature and allows for future extensions to non-triangular meshes. The GUI provides a simple way to use our approach. It allows users to load the mesh of the tumor and the organ, computes the CSA, provides a 3D visualization of the CSA over the two original models, and returns additional statistics such as the overall tumor area and volume. The source code for the GUI has ben publicly available on previously referenced GitHub repository. Figure 11 shows a screenshot of the output from our GUI, displaying an example of synthetic models. From the GitHub repository, it is also possible to download an executable of the GUI, making it easier for practitioners to adopt the approach.

This study received approval from the ethics committee of Policlinico San Martino, Genoa, Italy (Ethics Committee code: PT44; regional number: 554/2023).

## 4. Discussion

*Novelty of the Approach.* The approach proposed in this study provides a formalization of the computation of the CSA when accurate 3D models of an organ and tumor are available. The main novelty of our approach is that, contrary to previous solutions, it does not require assumptions about the tumor’s shape or manual annotations. Since the algorithm for computing the CSA uses the geometries of both the organ and the tumor, it is fundamental for the reconstruction to be as accurate as possible. In the article, we present a protocol to follow, which we executed using the software Materialise. However, alternative medical 3D reconstruction software could also be adopted. Although our work has been designed and tested for kidney tumors, we want to point out that the algorithm described here is general and could be applied to other organs. However, this remains a theoretical possibility that requires further validation through dedicated studies on different anatomical structures.

*Comparison with State-of-the-Art Solutions.* Our approach aims to reduce the influence of human error in the computation of the CSA. State-of-the-art solutions rely on values that must be manually measured by a human operator, which can decrease the accuracy of the CSA estimate and reproducibility of studies. Even methods based on 3D reconstructions require the manual annotation of the model to define the CSA, making it hard to reproduce calculations. Although our approach still requires human labor for creating 3D reconstructions, human intervention is only necessary for that step, not for identifying the CSA. Notably, the fact that the total tumor areas computed by our algorithm match those calculated by the Materialise software at the end of the reconstruction confirms that the algorithm performs highly precise and reliable calculations based on the 3D geometric structure of the object. Moreover, researchers are already working on automating organ and tumor segmentation from CT scans for 3D reconstructions, and a few commercial products are available for specific organs, such as the heart, with Materialise Mimics’ Heart Tool as an example. Therefore, in the foreseeable future, when 3D reconstructions can be autonomously executed, our approach will facilitate the computation of the CSA without requiring human intervention.

*Results Interpretation and Validation.* We evaluated our approach using both synthetic and real 3D models. For the synthetic test, where ground truth values were available, we estimated the percentage error and it resulted to be close to zero. The outliers observed in the boxplot result from the fact that the synthetic data were specifically designed to test the robustness of the algorithm, including cases with extreme and biologically improbable tumor shapes. Furthermore, the synthetic data we used for our evaluation is publicly available and can be used in future studies to evaluate new CSA computation approaches. Regarding the quantitative measurements obtained from the reconstructions of organs and tumors, the variation compared to the expert’s measurements is minimal. The results are very similar to those of the novice, although the novice’s measurements exhibit a lower variance, indicating that they are closer to the expert’s values than those obtained by our algorithm. However, it is important to note that these results are not based on ground truth values, but instead rely on the assumption that the expert’s measurements serve as the most reliable reference. Nevertheless, our results remain very close to the expert’s, both in terms of mean and median, with the presence of outliers comparable to that of the novice. This was further supported by the high correlation observed between the measurement distributions. To ensure maximum reusability of our results, our code is available as open-source software, and to facilitate ease of use, we also provide a graphical user interface that allows CSA computation from two STL files.

*Advantages and Limitations.* Our approach introduces several advantages. Unlike previous methods that rely on 2D images, our model is based on 3D structures, allowing for a more accurate and geometrically precise assessment of the tumor–organ interface. Furthermore, the CSA computation algorithm provides an objective and reliable measurement, as confirmed by the results obtained with the synthetic dataset. Another key strength of our method is the integration of a graphical user interface, which makes it accessible to users who need to apply this model without requiring extensive technical expertise.

However, some limitations remain. The accuracy of the segmentation process is highly dependent on the quality of the CT acquisition, which can introduce difficulties in obtaining clear and well-defined 3D reconstructions. To address this, our algorithm refines CSA calculations, as described in Section 2.2, identifying contact areas that may not be initially recognized through algorithmic correction. Additionally, since 3D reconstructions rely on manual or semi-automated segmentation, the quality of the final output may vary depending on the operator’s expertise.

## 5. Conclusions

This study introduces a novel method for calculating the CSA from 3D reconstructions of kidneys and tumors. By eliminating geometric assumptions and manual intervention in CSA computation, our approach enhances accuracy and reproducibility. However, the 3D reconstructions of the kidney and tumors are still performed manually in a semi-automatic way. To address this, we provide a segmentation protocol to ensure the reconstructions are as accurate as possible, while the CSA computation itself remains fully automated. We validated our approach using both synthetic data and 3D reconstructions of tumors and organs from patients. The results demonstrated the reliability of our method, as the CSA measurements obtained from the 3D reconstructions closely matched those made by an experienced radiologist. While automatic segmentation remains an area for future improvement, our approach offers a more objective and precise assessment of contact surface area measurement.

## Data Availability

Due to privacy concerns, the data related to patients cannot be shared. However, data pertaining to the algorithm code and synthetic data are available in the repository inserted in the manuscript accessible to all: https://github.com/ACarfi/contact-surface-area-gui, 3 March 2025.

## Supplementary Materials

The following supporting information can be downloaded at https://www.mdpi.com/article/10.3390/jimaging11030078/s1: Algorithm S1: A novel method to compute the contact surface area between an organ and cancer tissue. In the supplementary file, we have included all the formulas and the algorithm necessary for the calculation of the CSA that we presented in Section 2.2.

## Abbreviations

The following abbreviations are used in this manuscript:

CSA	Contact Surface Area
CT	Computed Tomography
DICOM	Digital Imaging and Communications in Medicine
HU	Hounsfield Unit
