# Supplementary material for: A Novel Method to Compute the Contact Surface Area Between an Organ and Cancer Tissue
Source: J Imaging. 2025 Mar 6;11(3):78. doi: 10.3390/jimaging11030078 (PMC11942950; doi:10.3390/jimaging11030078)
Supplement: Supplementary file 1 [file jimaging-11-00078-s001.zip › jimaging-3470830-supplementary.pdf]

Supplementary Materials: A novel method to compute the contact surface area between an organ and cancer tissue

All the formulas used and the algorithm developed for calculating the contact area are given below in a compact form. The mesh in 3D objects is composed by a number N of faces:

$$PM = \{F_1 \dots F_j \dots F_N\}. \tag{S1}$$

A face, according to its shape, is characterized by a set of M vertices, such as

$$F_j = \{V_{j1} \dots V_{jk} \dots V_{jM}\}, \tag{S2}$$

where each vertex is defined in the 3D Cartesian space as

$$V_{jk} = \{V_{jk}.x, V_{jk}.y, V_{jk}.z\}. \tag{S3}$$

Our approach for computing the CSA between two objects, O_1 (for example, the organ) and O_2 (for example, the tumour), is based on the following conceptual position. We assume that each object O_i with $i \in [1, 2]$, is described by a PM_i, which is composed of N_i planar faces, as

$$PM_i = \{F_{i1}, \dots F_{ij}, \dots F_{iN_i}\}. \tag{S4}$$

Each face F_{ij} is described by a ordered set of vertices of dimension M_{ij}

$$F_{ij} = \{V_{ij1} \dots V_{ijk} \dots V_{ijM_{ij}}\}, \tag{S5}$$

and each vertex is expressed in 3D Cartesian coordinates. Starting from that, we implemented the following algorithm.

Algorithm S1

Phase 1 - Computation of centroids

for each $PM_i \in \{PM_1, PM_2\}$ **do**
    for j=1 to N_i **do**
        $x \leftarrow 0$
        $y \leftarrow 0$
        $z \leftarrow 0$
        for k=1 to M_{ij} **do**
            $x \leftarrow x + V_{ijk}.x$
            $y \leftarrow y + V_{ijk}.y$
            $z \leftarrow z + V_{ijk}.z$
        end for
        $C_{ij}.x \leftarrow x / M_{ij}$
        $C_{ij}.y \leftarrow y / M_{ij}$
        $C_{ij}.z \leftarrow z / M_{ij}$
    end for
end for

Phase 2 - Computation of the centroid-to-centroid distance

```
p ← argmin(N_1, N_2)
q ← argmax(N_1, N_2)
for i=1 to N_p do
    for j=1 to N_q do
        Dt_j ← euclidean_distance(C_pi, C_qj)
    end for
    D_i ← min(Dt)
end for
```

Phase 3 - Computation of the threshold

```
Dt ← quicksort(D)
F ← 0
for i=1 to L do
    if Dt_i < 1 cm then
        Ds_i ← Dt_i
        F ← F + 1
    else
        break
    end if
end for
for i=2 to F − 1 do
    f1 ← lsq_fit(Ds_[1,i])
    f2 ← lsq_fit(Ds_[i+1,F])
    D̄_[1,i] ← f1(1, i)
    D̄_[i+1,F] ← f2(i + 1, F)
    d_i ← 0
    for j=1 to F do
        d_i ← d_i + euclidean_distance(Ds_j, D̄_j)
    end for
end for
id = argmin(d)
τ = Ds_id
```

Phase 4 - Refinement of the CSA

```
IDs ← {}
j ← 1
for i=1 to L do
    if D_i < τ then
        IDs_j ← i
        j ← j + 1
    end if
end for
```

Phase 5 - Refinement of the CSA

```
for i=1 to L do
    if i ∉ IDs then
        for k=1 to M_pi-1 do
            G.add_edge(V_pik, V_pi(k+1))
        end for
        G.add_edge(V_piM_pi, V_pi0)
    end if
end for
if not isconnected(G) then
    Sm ← connected_components(G)
end if
t ← {0}
for i=1 to |Sm| do
    for j=1 to |Sm_i| do
        if t_i ≤ D_Sm_ij then
            t_i ← D_Sm_ij
        end if
    end for
end for
not_csa = argmax(t)
for i=1 to |Sm| do
    if j ≠ not_csa then
        for j=1 to |Sm_i| do
            k ← length(IDs)
            IDs_k+1 ← j
        end for
    end if
end for
```

Phase 6 - Computation of the CSA

```
CSA ← 0
for i=1 to |IDs| do
    j ← IDs_i
    CSA ← CSA + area(C_pj)
end for
```

Then to compute the toal area of CSA, we used the following formula:

$$A = \frac{1}{2}\left|\sum_{i=1}^{n-1} x_i y_{i+1} + x_n y_1 - \sum_{i=1}^{n-1} x_{i+1} y_i - x_1 y_n\right|. \quad \text{(S6)}$$
